# Supplementary material for: YAP-independent mechanotransduction drives breast cancer progression
Source: Nat Commun. 2019 Apr 23;10:1848. doi: 10.1038/s41467-019-09755-0 (PMC6478686; doi:10.1038/s41467-019-09755-0)
Supplement: Supplementary file 1 — Supplementary Information [file 41467_2019_9755_MOESM1_ESM.pdf]

Supplementary Information

YAP-independent mechanotransduction drives breast cancer progression

Joanna Y. Lee[1], Jessica Chang[2], Antonia A. Dominguez[3-5], Hong-pyo Lee[1], Sungmin Nam[1], Julie Chang[3], Sushama Varma[6], Lei S. Qi[3-5], Robert B. West[6] and Ovijit Chaudhuri[1]*

Affiliations:

[1]Department of Mechanical Engineering, Stanford University, Stanford, CA 94305.

[2]Department of Genetics, Stanford University School of Medicine, Stanford, CA 94305.

[3]Department of Bioengineering, Stanford University, Stanford, CA 94305.

[4]Department of Chemical and Systems Biology, Stanford University, Stanford, CA 94305.

[5]Stanford ChEM-H, Stanford University, Stanford, CA 94305.

[6]Department of Pathology, Stanford University School of Medicine, Stanford, CA 94305.

*Correspondence to: chaudhuri@stanford.edu

Includes:
Supplementary Figures 1–14
Supplementary Tables 1 and 2

Supplementary Figures

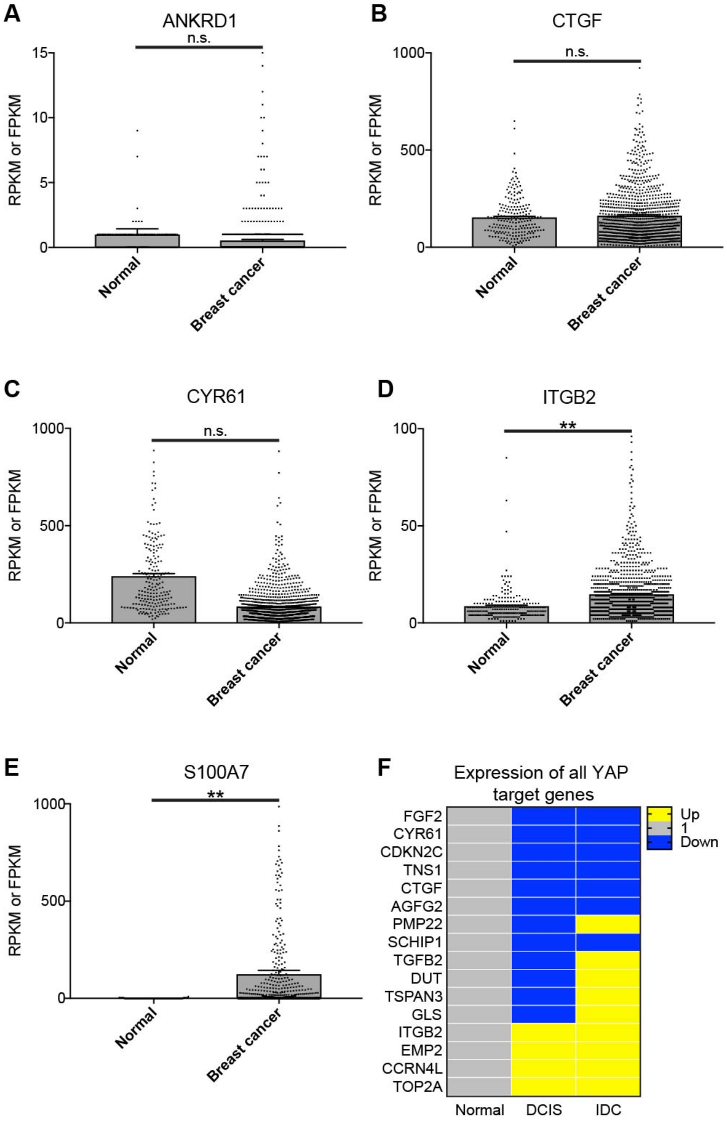

Supplementary Figure 1. Expression of YAP target genes in breast cancer patient datasets. RNA-seq gene expression counts of canonical YAP target genes **(A)** ANKRD1, **(B)** CTGF, **(C)** CYR61, **(D)** ITGB2, and control **(E)** S100A7, in normal mammary and breast cancer tissues obtained from Human Protein Atlas[1,2]. Normal and breast cancer results are reported as RPKM (GTEx dataset) and FPKM (TCGA dataset), respectively. (n.s., $p > 0.05$; **, $p < 0.05$, one-way ANOVA followed by Tukey post-hoc comparison tests, n = 214-1075 patient samples, error bars represent SEM, symbols represent each patient sample, some symbols beyond graph axes). **(F)** Diagram showing increased or decreased expression of YAP target genes during breast cancer progression from Brunner *et al*. study[3].

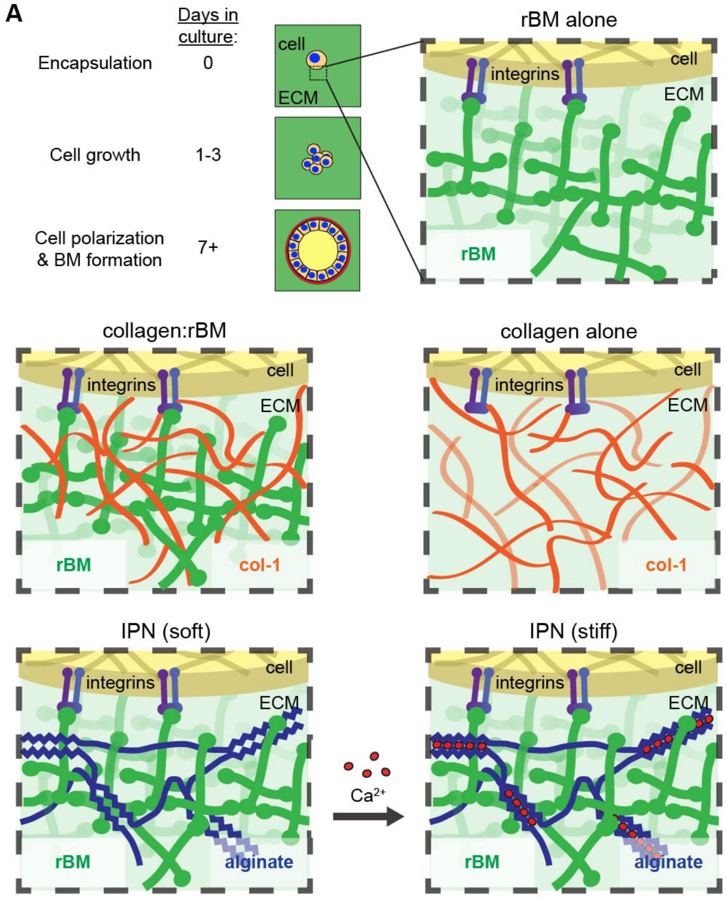

B

Hydrogel	rBM (mg/ml)	col (mg/ml)	alg (%)	Ca2+ (mM)	E (kPa)
Soft rBM	4.4				0.07
Soft col:rBM	4.4	0.5			0.07
Med. col:rBM	4.4	3			0.3
Soft col		3			0.1
Stiff col		10			1.1
Soft IPN	4.4		0.5	0	0.04
Stiff IPN	4.4		0.5	20	1.9

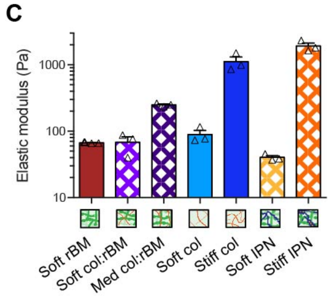

Supplementary Figure 2. Generation of hydrogels with range of elastic moduli and col-1 concentration. (A) Schematic of hydrogel encapsulated MCF10A forming an acinar structure and hydrogels generated in this study. rBM: reconstituted Basement Membrane; col-1: type 1 collagen. **(B)** Composition and stiffness of hydrogels used in this study. alg: alginate; col: col-1; E: elastic modulus. **(C)** Elastic modulus measurements of hydrogels at 1 Hz. Bars represent mean of three gels ± SEM, symbols represent E of each gel.

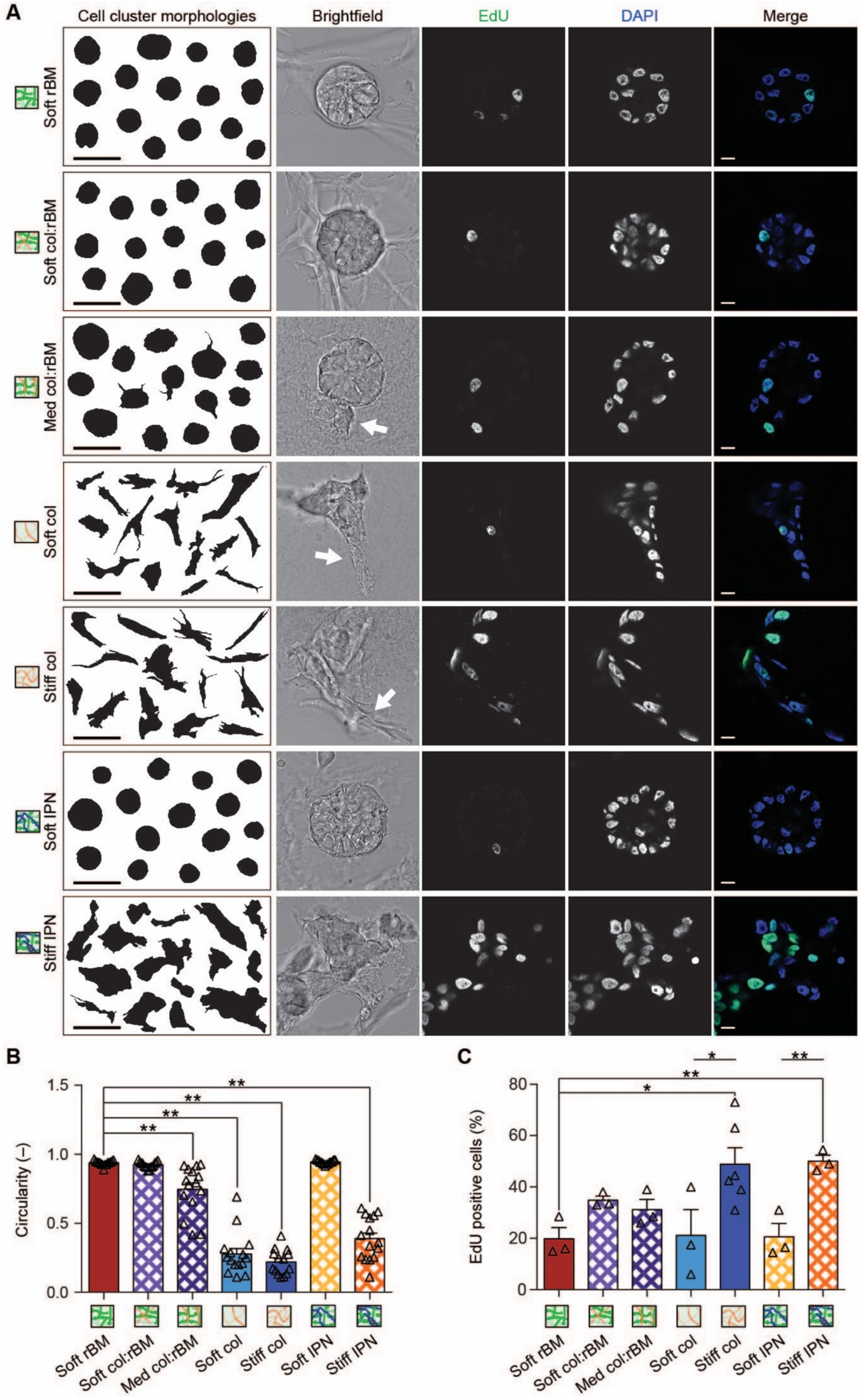

Supplementary Figure 3. Enhanced ECM stiffness promotes invasion and proliferation in 3D culture in the presence or absence of col-1. (A) MCF10A cells encapsulated for seven days, with 24 h EdU treatment, were fixed and stained for EdU (green). DNA was stained using DAPI (blue), scale bars: 10 µm. Arrows denote invasive structures. Morphological analysis of 14-15 cell clusters from each hydrogel are displayed in first column, scale bars: 100 µm. **(B)** Invasiveness of cells in each hydrogel as measured by cell cluster circularity (**, $p < 0.001$, one-way ANOVA followed by Tukey post-hoc comparison tests, n = 14 cell clusters for stiff col, n = 15 cell clusters for all others, from 1 experiment, error bars represent SEM, symbols represent each cell cluster). **(C)** Graph of percent EdU positive cells in each hydrogel. (**, $p < 0.05$; *, $p < 0.1$, one-way ANOVA followed by Tukey post-hoc comparison tests, n = 200 cells per experiment, from 3 experiments for all except stiff col which had 6 experiments, error bars represent SEM, symbols represent mean of each experiment).

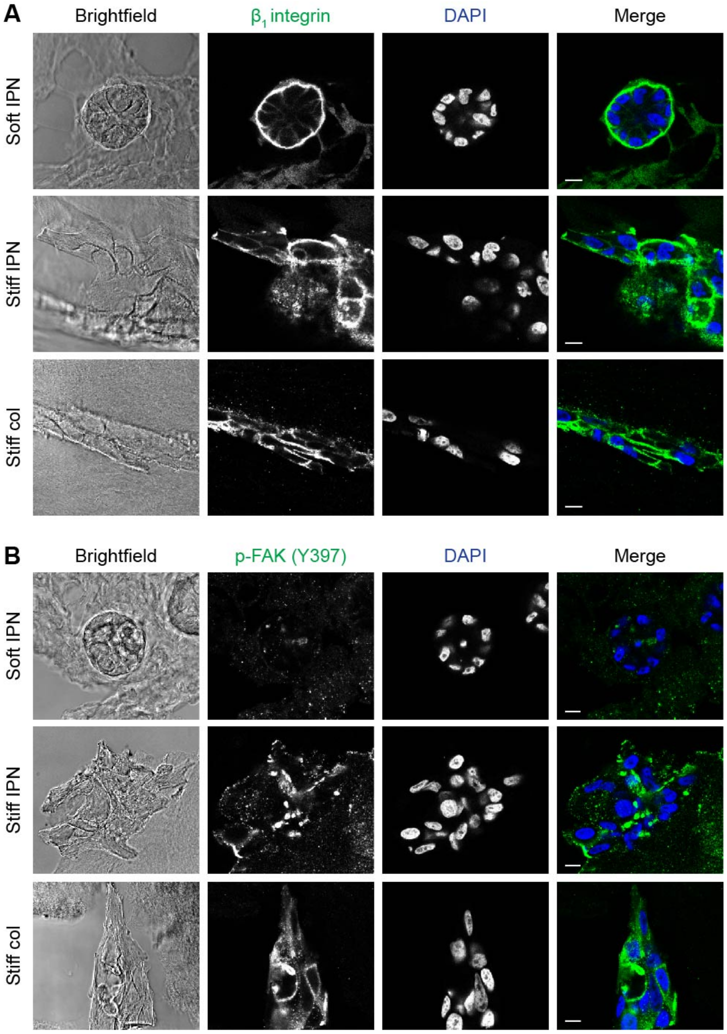

Supplementary Figure 4. Assessment of general mechanotransduction pathways. Confocal micrographs of MCF10A cells encapsulated for seven days in indicated hydrogels and stained for **(A)** β_1 integrin (green) or **(B)** phospho-FAK (Y397) (green). DNA is stained using DAPI (blue). Scale bars: 10 µm.

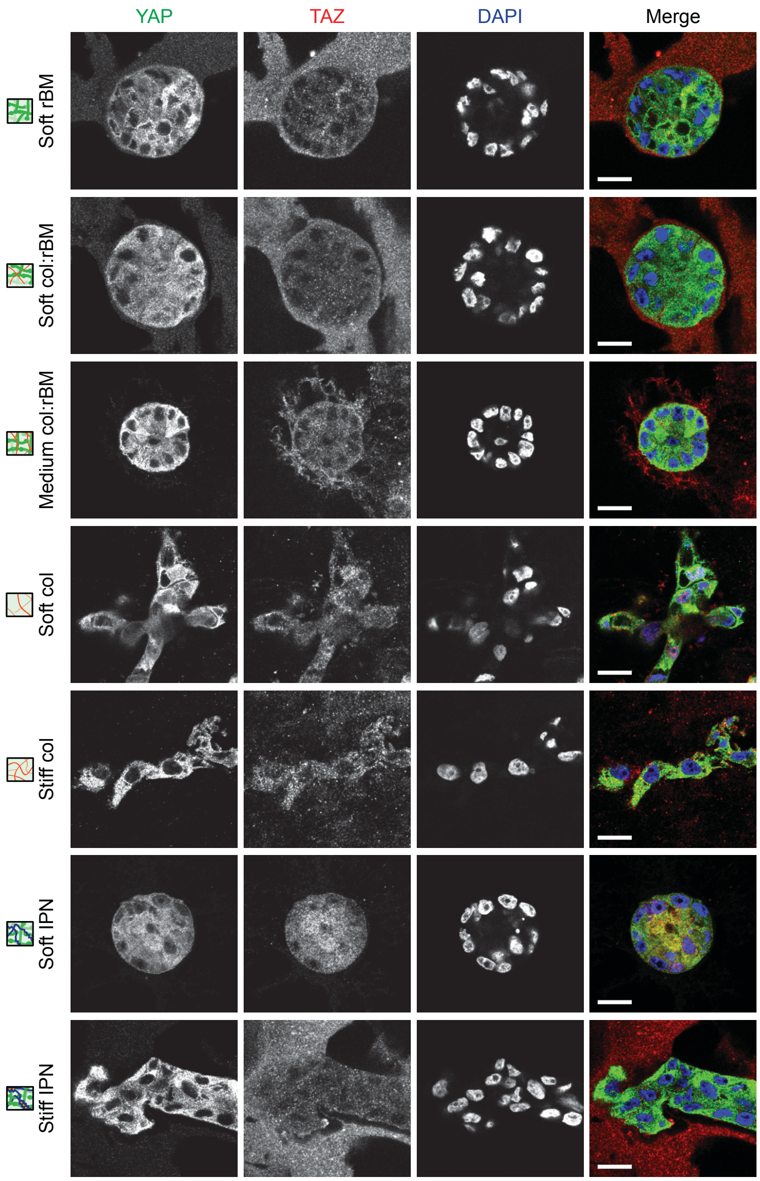

Supplementary Figure 5. TAZ localization. Confocal micrographs of MCF10A cells encapsulated for seven days in indicated hydrogels corresponding to Fig. 2 stained for YAP (green), TAZ (red), and DNA using DAPI (blue). Scale bars: 10 µm. We note there is also ECM staining by the TAZ antibody.

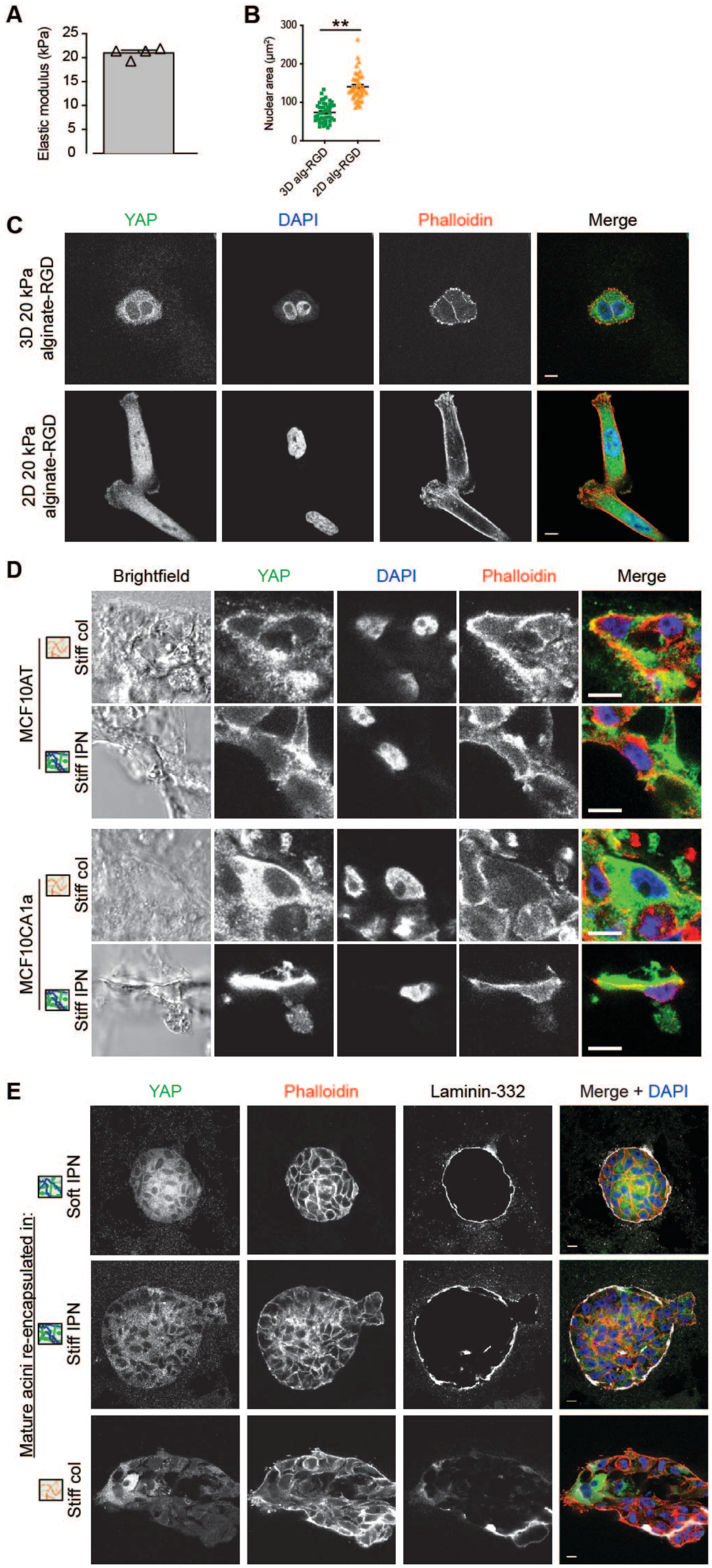

Supplementary Figure 6. YAP staining with 20 kPa stiffness, MCF10AT and MCF10CA1a cells, and transplantations. (A) Unconfined compression of hydrogels compressed to 15% at a rate of 1 mm min^-1 to obtain the stiffness of alginate hydrogels. Bars represent mean of four gels ± SEM, symbols represent E of each gel. **(B)** Nuclear areas of MCF10A cells cultured under 2D and 3D conditions with 20 kPa alginate-RGD. Lines display mean ± SEM. **, $p < 0.0001$; unpaired t-test. **(C)** MCF10A cells encapsulated in 3D for 3 days or in 2D for 1 day using 20 kPa alginate-RGD hydrogels. Scale bars: 10 µm. **(D)** MCF10AT and MCF10CA1a cells encapsulated for seven days. YAP (green). F-actin was stained with phalloidin (red) and DNA with DAPI (blue). Scale bars: 10 µm. **(E)** Mature MCF10A acini harvested from seven day rBM overlay cultures[4] and then encapsulated in stiff IPNs for three days. Cells were stained for YAP (green), F-actin using phalloidin (red), laminin-332 (white), and DNA using DAPI (blue). Scale bars: 10 µm.

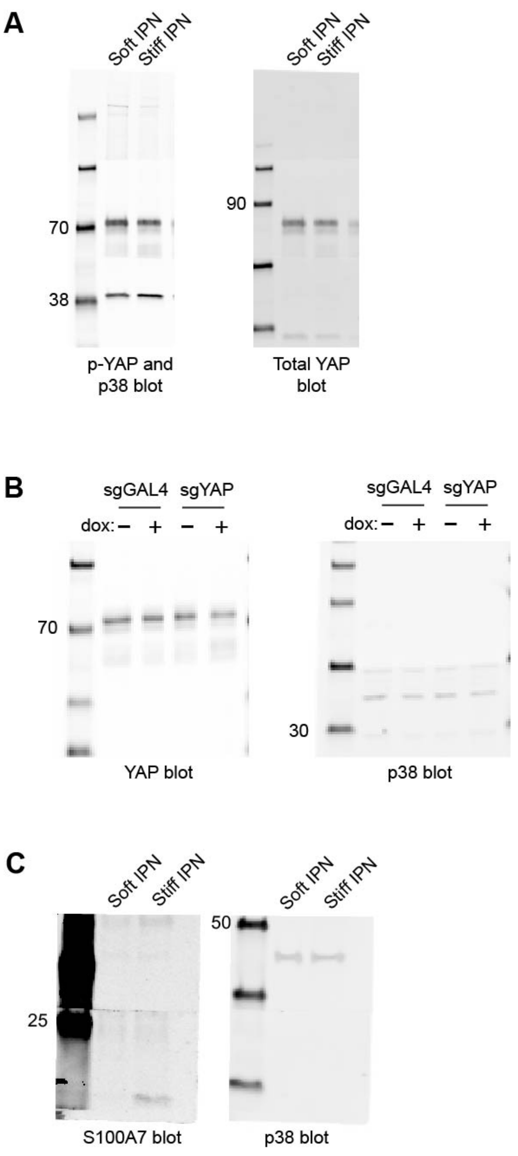

Supplementary Figure 7. Uncropped Western blots. **(A)** p-YAP, total YAP, and p38 in IPNs. **(B)** YAP and p38 in GAL4 and YAP knockout cells. **(C)** S100A7 in IPNs.

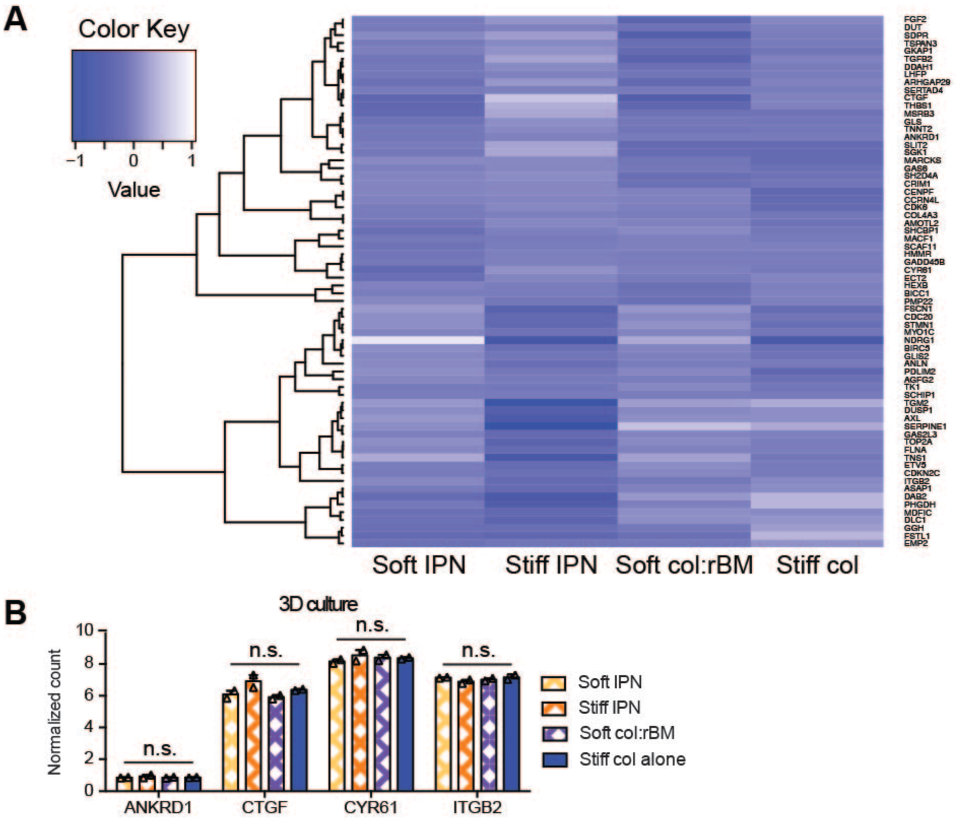

Supplementary Figure 8. Enhanced col-1 stiffness does not promote YAP target gene expression. (A) Expression of YAP target genes (as identified by Dupont et al., 2011) in MCF10A cells encapsulated in indicated hydrogels for seven days and analyzed by RNA-seq. Values are normalized by mean of each row. **(B)** Graph of RNA-seq normalized counts of canonical YAP target genes in each hydrogel. (n.s., $p > 0.05$; one-way ANOVA followed by Tukey post-hoc comparison tests, n = 2 independent experiments, error bars represent S.D., symbols represent each experiment).

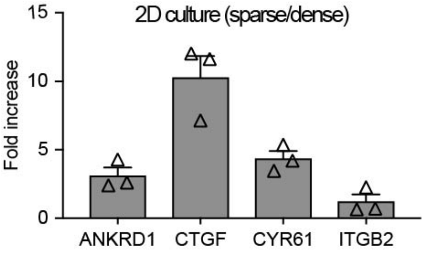

Supplementary Figure 9. YAP target gene expression in 2D culture. Graph of expression fold change of canonical YAP target genes with YAP activating (2D sparse plating) over YAP inactivating (2D dense plating) conditions in MCF10A cells. (n = 3 samples from 1 experiment, error bars represent SEM, symbols represent each replicate).

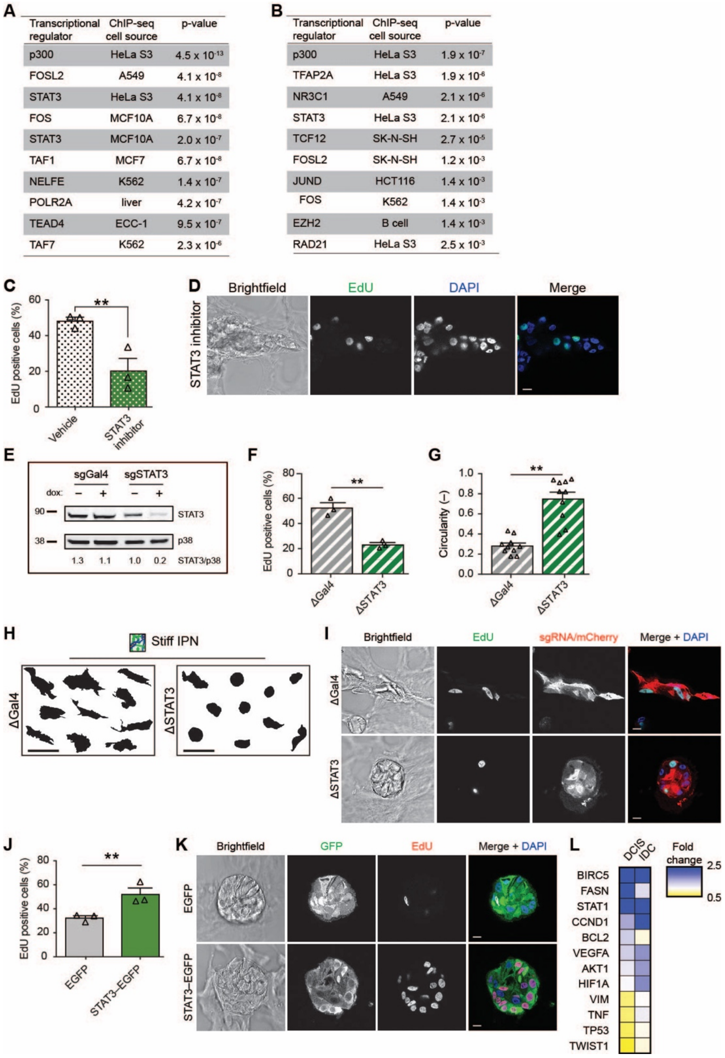

Supplementary Figure 10. STAT3-dependent mechanotransduction in 3D culture. Table of transcriptional regulators most significantly associated with DE genes induced by increased **(A)** IPN stiffness (stiff IPN/soft IPN) or **(B)** col-1 stiffness (stiff col/soft col:rBM), as defined by ChIP-seq data provided by ENCODE. **(C)** Graph of percent EdU positive MCF10A cells encapsulated for seven days in stiff IPNs and treated for three days with 500 μM STAT3 peptide inhibitor PY*LKTK or vehicle (DMSO). (**, $p < 0.05$, one-way ANOVA followed by Tukey post-hoc comparison tests, n = 200 cells per experiment, from 3 experiments, error bars represent SEM, symbols represent mean of each experiment). **(D)** Confocal images of encapsulated MCF10A cells from (C) stained for EdU (green) and DNA using DAPI (blue). Scale bars: 10 μm. **(E)** Western blot analysis of MCF10A::Cas9/sgGAL4 or MCF10A::Cas9/sgSTAT3 cells with or without 2 μg/ml dox for 72 h and probed for STAT3. p38 was used as a loading control. Fluorescence intensity quantification of bands, normalized by p38 control, are indicated below each lane. **(F)** Graph of percent EdU positive cells in each hydrogel. (**, $p < 0.01$, one-way ANOVA followed by Tukey post-hoc comparison tests, n = 200 cells per experiment, from 3 experiments, error bars represent SEM, symbols represent mean of each experiment). **(G)** Invasiveness of cells in each hydrogel as measured by cell cluster circularity, (**, $p < 0.01$, one-way ANOVA followed by Tukey post-hoc comparison tests, n = 10 cell clusters, from 1 experiment, error bars represent SEM, symbols represent each cell cluster). **(H)** Cell cluster morphologies of ΔGAL4 (control) or ΔSTAT3 MCF10A cells encapsulated for seven days with 2 μg/ml dox in stiff IPNs. Scale bars: 100 μm. **(I)** Representative cluster from (H) stained for EdU (green), sgRNA/mCherry (red), and DNA using DAPI (blue). Scale bars: 10 μm. **(J)** Graph of percent EdU positive MCF10A cells transduced with STAT3-EGFP or EGFP control and encapsulated for seven days in stiff IPNs from (**, $p < 0.01$, one-way ANOVA followed by Tukey post-hoc comparison tests, n = 200 cells per experiment, from 3 experiments, error bars represent SEM, symbols represent mean of each experiment). **(K)** Representative image from (J) stained for GFP (green), EdU (red), and DNA using DAPI (blue). Scale bars: 10 μm. **(L)** Heat map showing expression of selected STAT3 target genes that are highly relevant to cancer in DCIS or IDC patient samples. Values are displayed as fold change compared to normal.

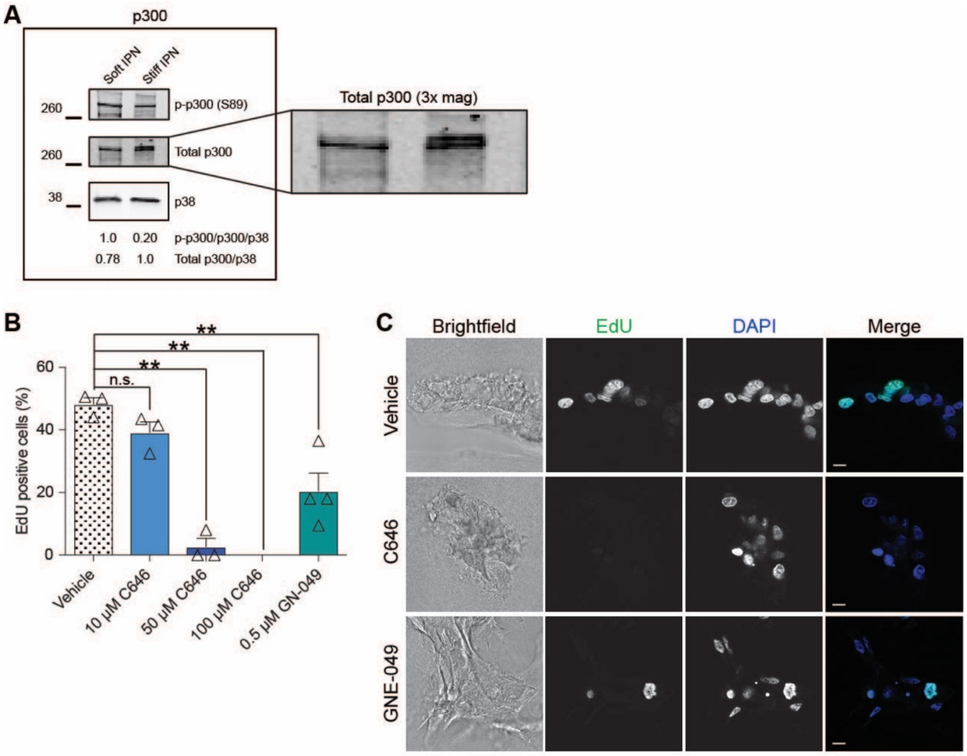

Supplementary Figure 11. p300 stiffness-induced gene regulation. (A) Western blot analysis of cells harvested from soft and stiff IPNs and probed for p-p300 (S89) and total p300 (with 3x magnification) p38 was used as a loading control. Fluorescence intensity quantification of bands, normalized by p38 control, are indicated below each lane. **(B)** Graph of percent EdU positive MCF10A cells encapsulated for seven days in stiff IPNs and treated for three days with p300 inhibitors C646 or GNE-049 or vehicle (DMSO). (n.s., $p > 0.05$; **, $p < 0.05$, one-way ANOVA followed by Tukey post-hoc comparison tests, n = 200 cells per experiment, from 3 experiments, error bars represent SEM, symbols represent mean of each experiment). **(C)** Confocal images of 100 µM C646 or 0.5 µM GNE-049 treated cells from (e) stained for EdU (green) and DNA using DAPI (blue). Scale bars: 10 µm.

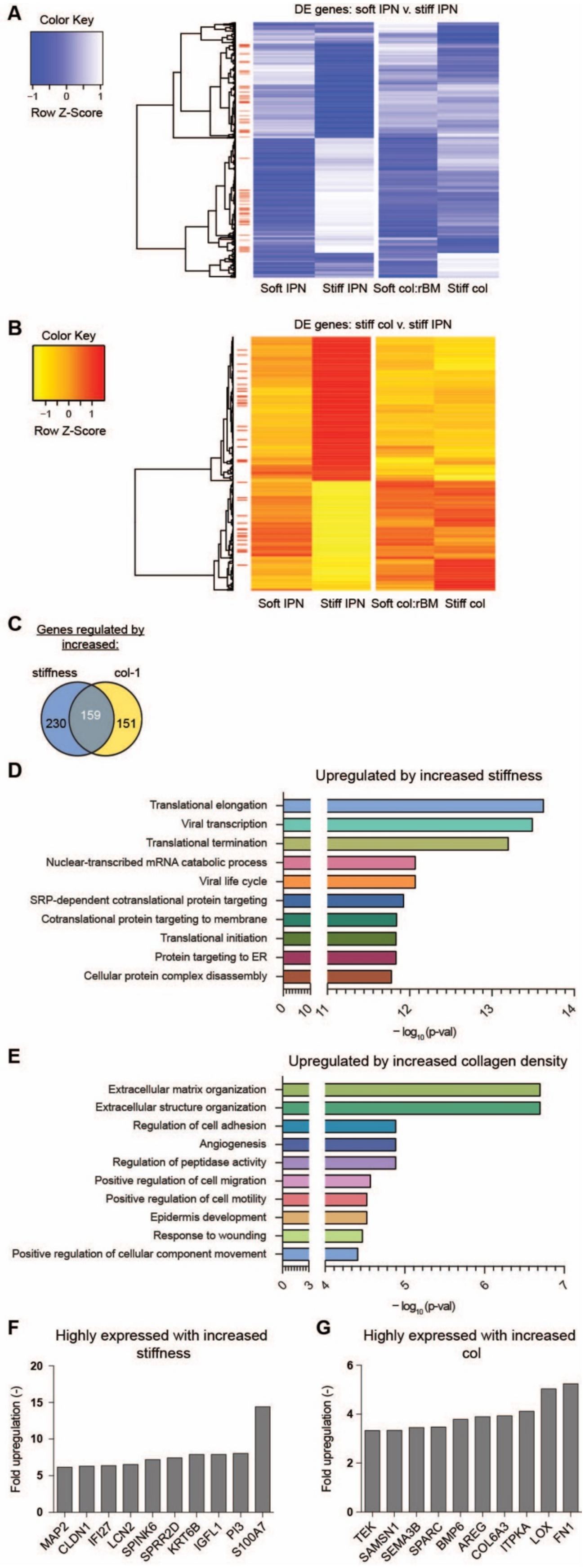

Supplementary Figure 12. RNA-seq analysis reveals alteration in gene expression of MCF10A cells due to increased stiffness or enhanced collagen density in 3D culture. (A) Heat map of 389 genes differentially expressed (DE) at FDR < 0.05 when comparing MCF10A cells harvested from soft v. stiff IPNs. Red bars denote genes involved in proliferation, cell cycle, mitosis, or adhesion. Values are displayed as row Z-scores. **(B)** Heat map of 310 genes DE at FDR < 0.05 when comparing stiff IPNs v. stiff col-1 hydrogels. **(C)** Venn diagram showing number of genes regulated by both enhanced stiffness and enhanced col-1 density. **(D)** Bar graph of gene ontology (GO) terms significantly represented in differentially expressed genes during increased stiffness (stiff IPN/soft IPN) or **(E)** increased col-1 (stiff col/stiff IPN). **(F)** Bar graph of most highly upregulated genes during increased stiffness or **(G)** increased col-1.

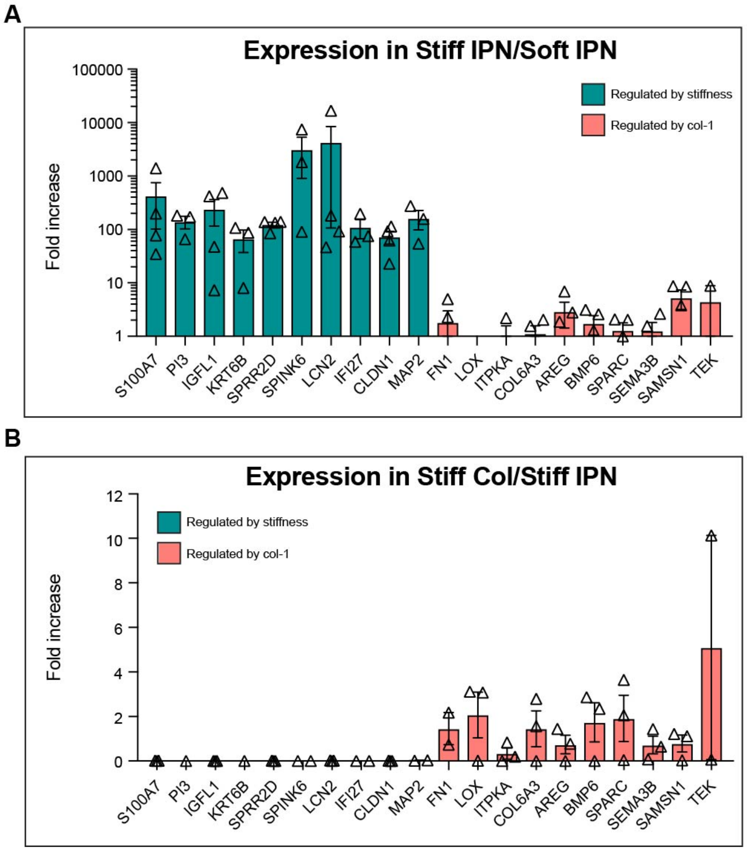

Supplementary Figure 13. qPCR validation of 3D culture RNA-seq. **(A)** Graph showing qPCR results of RNA-seq identified stiffness- and col-regulated genes in 3D cultured stiff IPNs (compared to soft IPNs). **(B)** Graph showing qPCR results of RNA-seq identified stiffness- and col-regulated genes in 3D cultured stiff col-1 hydrogels (compared to stiff IPNs). (n = 3 experiments, error bars represent SEM, symbols represent each experiment).

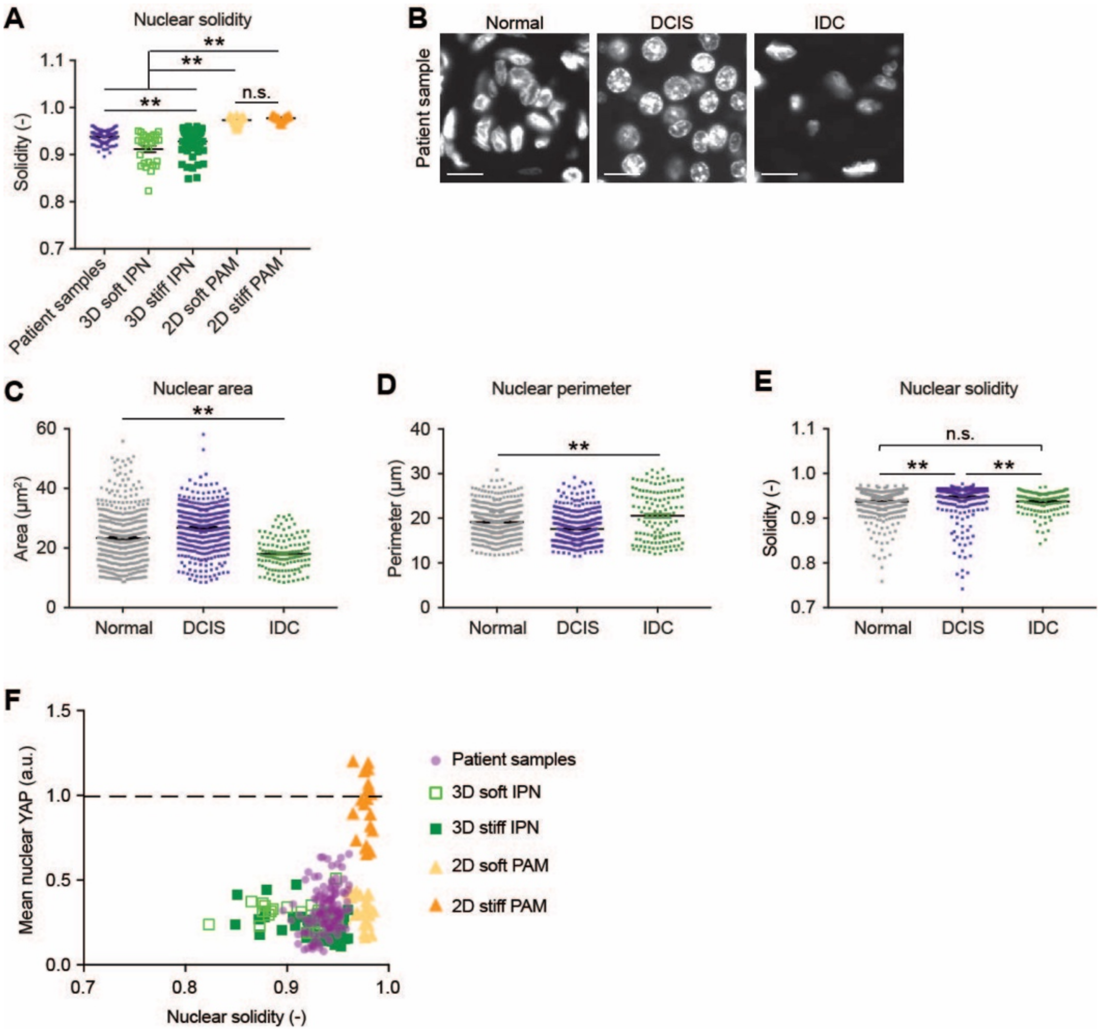

Supplementary Figure 14. Nuclear solidity and morphology changes during *in vivo* cancer progression. (A) Solidity of nuclei (value of 1 represents a perfect circle). Patient samples from five DCIS patients. **(B)** Images of nuclear morphologies during breast cancer progression. Bars: 10 μm. **(C)** Areas, **(D)** perimeters, and **(E)** solidity of nuclei. Patient samples from five normal, 5 DCIS, and 5 IDC patients. **(F)** YAP intensity with nuclear solidity. Bar charts display mean ± SEM. **, $p < 0.05$; one-way ANOVA followed by Tukey post-hoc comparison tests.

Supplementary Tables

AMOTL2	CDKN2C	DUSP1	GAS2L3	LHFP	SCHIP1	TGFB2
ANKRD1	CENPF	DUT	GAS6	MACF1	SDPR	TGM2
ANLN	COL4A3	ECT2	GGH	MARCKS	SERPINE1	THBS1
ARHGAP29	CRIM1	EMP2	GKAP1	MDFIC	SERTAD4	TK1
AXL	CTGF	ETV5	GLIS2	MSRB3	SCAF11	TNNT2
BICC1	CYR61	FGF2	GLS	MYO1C	SGK1	TNS1
BIRC5	DAB2	FLNA	HEXB	NDRG1	SH2D4A	TOP2A
CCRN4L	DDAH1	FSCN1	HMMR	PDLIM2	SHCBP1	TSPAN3
CDC20	ASAP1	FSTL1	AGFG2	PHGDH	SLIT2	
CDK6	DLC1	GADD45B	ITGB2	PMP22	STMN1	

Supplementary Table 1. YAP target genes used in Fig. 2F.

	Fold change (Stiff IPN/Soft IPN)	Fold change (DCIS/Norm)
S100A7	14.41	168.6296296
CEACAM6	3.56	17.2380952
FOXA1	2.18	2.7468672
OVOL1	2.19	2.7568922
SPAG1	2.19	2.8531469
SAMD12	2.37	1.7521059
IVL	2.65	7.1111111
IGFL1	7.91	5.4285714
FAM84B	1.81	1.6358543
HES2	2.69	6.5641026
SAMD9	2.33	2.3884058
IL1RN	2.15	2.5824783
MAL2	2.77	2.2711542
ANO9	2	1.9725408
PGBD5	3.55	2.3670412
NQO1	1.79	2.2589532
IFI6	2.58	12.61816
HMGCS1	2.96	1.6597837
CFB	2.03	2.7285044
LLGL2	2.31	1.8260076
LEPREL1	0.45	0.256596
PRX	0.49	0.4746312
MME	0.52	0.3169467
GLTSCR2	0.57	0.3878207
CHST2	0.48	0.3195779
RPL13	0.6	0.3437348
SRPX	0.47	0.1300813
ICAM5	0.43	0.2816901
LRP1	0.48	0.3622555
LAMB1	0.57	0.3884762
PTPRS	0.57	0.4469853
SEMA4G	0.46	0.4638562
RPS14	0.59	0.3880393
LOC100506548	0.51	0.5722983
COL17A1	0.58	0.3116743
TMEM176B	0.4	0.3407196
DNAJB5	0.51	0.3501199
RPS5	0.57	0.4548077
GNB2L1	0.53	0.4325783
IRS2	0.37	0.5308699
CSRNP1	0.51	0.3459834
TXNIP	0.34	0.5108061
RPL4	0.59	0.4429762
ITGB4	0.43	0.5625764
PDPN	0.53	0.4311068
RPS15	0.61	0.5901639
RPS11	0.59	0.4160321
AXL	0.52	0.5741028

Supplementary Table 2. Genes with similar regulation in stiff IPNs as DCIS samples in Fig. 2L.

Supplementary references

1. Uhlén, M. *et al.* Proteomics. Tissue-based map of the human proteome. *Science* **347,** 1260419 (2015).
2. Uhlén, M. *et al.* Towards a knowledge-based Human Protein Atlas. *Nat. Biotechnol.* **28,** 1248–1250 (2010).
3. Brunner, A. L. *et al.* A shared transcriptional program in early breast neoplasias despite genetic and clinical distinctions. *Genome Biol.* **15,** R71 (2014).
4. Debnath, J., Muthuswamy, S. K. & Brugge, J. S. Morphogenesis and oncogenesis of MCF-10A mammary epithelial acini grown in three-dimensional basement membrane cultures. *Methods* **30,** 256–268 (2003).
