# Supplementary material for: YAP-independent mechanotransduction drives breast cancer progression
Source: Nat Commun. 2019 Apr 23;10:1848. doi: 10.1038/s41467-019-09755-0 (PMC6478686; doi:10.1038/s41467-019-09755-0)
Supplement: Supplementary file 3 — Reporting Summary [file 41467_2019_9755_MOESM3_ESM.pdf]

nature research

Corresponding author(s): Ovijit Chaudhuri

Last updated by author(s): Mar 27, 2019

Reporting Summary

Nature Research wishes to improve the reproducibility of the work that we publish. This form provides structure for consistency and transparency in reporting. For further information on Nature Research policies, see Authors & Referees and the Editorial Policy Checklist.

Statistics

For all statistical analyses, confirm that the following items are present in the figure legend, table legend, main text, or Methods section.

n/a	Confirmed	
☐	☒	The exact sample size (*n*) for each experimental group/condition, given as a discrete number and unit of measurement
☐	☒	A statement on whether measurements were taken from distinct samples or whether the same sample was measured repeatedly
☐	☒	The statistical test(s) used AND whether they are one- or two-sided<br>*Only common tests should be described solely by name; describe more complex techniques in the Methods section.*
☐	☒	A description of all covariates tested
☐	☒	A description of any assumptions or corrections, such as tests of normality and adjustment for multiple comparisons
☐	☒	A full description of the statistical parameters including central tendency (e.g. means) or other basic estimates (e.g. regression coefficient) AND variation (e.g. standard deviation) or associated estimates of uncertainty (e.g. confidence intervals)
☐	☒	For null hypothesis testing, the test statistic (e.g. *F*, *t*, *r*) with confidence intervals, effect sizes, degrees of freedom and *P* value noted<br>*Give P values as exact values whenever suitable.*
☒	☐	For Bayesian analysis, information on the choice of priors and Markov chain Monte Carlo settings
☒	☐	For hierarchical and complex designs, identification of the appropriate level for tests and full reporting of outcomes
☒	☐	Estimates of effect sizes (e.g. Cohen's *d*, Pearson's *r*), indicating how they were calculated

Our web collection on statistics for biologists contains articles on many of the points above.

Software and code

Policy information about availability of computer code

Data collection	Microscopy images were collected using LasX software (Leica). WBs visualized with the Li-COR Odyssey imaging system (Li-COR Biotechnology). RNA-seq data were collected using the Illumina Hiseq 2500 platform.
Data analysis	Photoshop CS6 (Adobe), Illustrator CS6 (Adobe), ImageJ 1.42q (NIH), Bowtie2 (Langmead and Salzberg, 2012, http://bowtie-bio.sourceforge.net/bowtie2/index.shtml), HTSeq (Anders, Pyl, and Huber, 2015, http://htseq.readthedocs.io/en/release_0.9.1/), DESeq2 (Huber et al., 2015 and Gentleman et al., 2014, http://bioconductor.org/packages/release/bioc/html/DESeq2.html)

For manuscripts utilizing custom algorithms or software that are central to the research but not yet described in published literature, software must be made available to editors/reviewers. We strongly encourage code deposition in a community repository (e.g. GitHub). See the Nature Research guidelines for submitting code & software for further information.

Data

Policy information about availability of data

All manuscripts must include a data availability statement. This statement should provide the following information, where applicable:

- Accession codes, unique identifiers, or web links for publicly available datasets
- A list of figures that have associated raw data
- A description of any restrictions on data availability

RNA-seq data are stored in GEO with the accession code GSE102506. 3SEQ breast cancer progression data are available as described in the original manuscript28. Additional normal mammary and breast cancer datasets generated by the Genotype-Tissue Expression (GTEx) project and The Cancer Genome Atlas (TCGA) project, respectively, are available on the Human Protein Atlas40,41. Additional datasets generated during and/or analysed during the current study are available from the corresponding author on reasonable request.

Field-specific reporting

Please select the one below that is the best fit for your research. If you are not sure, read the appropriate sections before making your selection.

☒ Life sciences ☐ Behavioural & social sciences ☐ Ecological, evolutionary & environmental sciences

For a reference copy of the document with all sections, see nature.com/documents/nr-reporting-summary-flat.pdf

Life sciences study design

All studies must disclose on these points even when the disclosure is negative.

Sample size	Sample size was determined following a similar study (Wei, et al., Nat Cell Biol, 2014). The authors used n = 50 cells, with 3 independent experiments. In this study we use n = 200 cells, with 3 independent experiments.
Data exclusions	No data were excluded.
Replication	All attempts at replication were successful.
Randomization	Study does not involve animals or human participants.
Blinding	Study does not involve animals or human participants.

Reporting for specific materials, systems and methods

We require information from authors about some types of materials, experimental systems and methods used in many studies. Here, indicate whether each material, system or method listed is relevant to your study. If you are not sure if a list item applies to your research, read the appropriate section before selecting a response.

Materials & experimental systems

n/a	Involved in the study	
☐	☒	Antibodies
☐	☒	Eukaryotic cell lines
☒	☐	Palaeontology
☒	☐	Animals and other organisms
☒	☐	Human research participants
☐	☒	Clinical data

Methods

n/a	Involved in the study	
☒	☐	ChIP-seq
☒	☐	Flow cytometry
☒	☐	MRI-based neuroimaging

Antibodies

Antibodies used	Mouse anti–YAP (cat. #sc-101199; Santa Cruz Biotech) was used at 1:200 (IF) and 1:500 (WB), rabbit anti–phospho-YAP (cat. #13008; Cell Signaling Technology) was used at 1:500 for WB. Mouse anti–S100A7 (cat. #sc-377084; Santa Cruz Biotech) was used at 1:500 (WB). Mouse anti-S100A7 (cat. #HPA006997; Millipore-Sigma) was used at 1:100 for IHC. Rabbit anti–phospho p300 (cat. #ab135554; Abcam) was used at 1:500 for WB, mouse anti–p300 (cat. #sc-32244; Santa Cruz Biotech) was used at 1:500 for WB. Rabbit anti–phospho STAT3 (cat. #ab76315; Abcam) was used at 1:500 for WB, mouse anti–STAT3 (cat. #sc-8019; Santa Cruz Biotech) was used at 1:500 for WB. Rabbit anti-p38 (cat. #sc-535; Santa Cruz Biotechnology) was used at 1:2000 for WB. Mouse anti-β1 integrin (cat. #ab24693, Abcam) was used at 1:500 for IHC. Rabbit anti-phospho FAK (cat. #31H5L17; Thermo Fisher Scientific) was used at 1:100 for IHC. Rabbit anti-α-actinin (cat. #3134; Cell Signaling Technology) was used at 1:500 for WB. Rabbit anti-β-actin (cat. #8457; Cell Signaling Technology) was used at 1:1000 for WB. Phalloidin-Alexa555 (A34055; Thermo Fisher Scientific) was used at 1:100 and DAPI (cat. #D9542; Sigma-Aldrich) was used at 5 µg/ml for IF.<br><br>For IF, Alexa 488-, 555- or 647-conjugated secondary antibodies (Thermo Fisher Scientific) were used at 1:500. For WB, IRDye 680 or 800-conjugated secondary antibodies (LI-COR Biotechnology) were used at 1:10,000.
Validation	Validations were performed as described on the manufacturers' websites. Additional validations were performed by immunofluorescence or western blotting on MCF10A cells.

Eukaryotic cell lines

Policy information about cell lines

Cell line source(s)	ATCC

Authentication	Cell line was not authenticated as it was directly obtained from the ATCC.
Mycoplasma contamination	Cell line was not tested for mycoplasma as it was directly obtained from the ATCC.
Commonly misidentified lines (See ICLAC register)	No commonly misidentified cell lines were used.

Clinical data

Policy information about clinical studies
All manuscripts should comply with the ICMJE guidelines for publication of clinical research and a completed CONSORT checklist must be included with all submissions.

Clinical trial registration	No clinical studies were performed.
Study protocol	No clinical studies were performed.
Data collection	No clinical studies were performed.
Outcomes	No clinical studies were performed.
